# Structural Basis for TRF2-RAP1 Recruitment by EBNA1 at the EBV origin of replication

**DOI:** 10.21203/rs.3.rs-7594663/v1

**Published:** 2025-10-29

**Authors:** Paul Lieberman, Samantha Sustek, Troy Messick, Jayaraju Dheekollu, Coltin Albitz, Christopher Chen, Anneliese Faustino, Hsin-Yao Tang, Hee Jong Kim, Kenji Murakami

**Affiliations:** The Wistar Institute; The Wistar Institute; The Wistar Institute; The Wistar Institute; The Wistar Institute; The Wistar Institute; The Wistar Institute; The Wistar Institute; University of Pennsylvania; University of Pennsylvania

**Keywords:** EBV, EBNA1, Shelterin, TRF2, hRAP1, TERF2, TERF2IP, CryoEM, oriP

## Abstract

Epstein-Barr Nuclear Antigen 1 (EBNA1) is essential for the episomal maintenance and DNA replication of Epstein-Barr virus (EBV) in latently infected cells and acts through binding to *oriP*. The minimal replicative unit of *oriP* (½DS) contains four EBNA1 binding sites flanked by single telomeric nonamers that recruit shelterin proteins TRF2 and Rap1, but the structural basis for host-factor engagement is not known. Here, we integrate cryo-electron microscopy, zero-length cross-linking mass spectrometry, Alphafold3 modeling, and biochemical binding assays to define the complex formed by EBNA1-TRF2-Rap1 assembly on the ½DS. We find that a highly dynamic complex is formed, with the TRF2 homodimerization domain (TRFH) flexibly interacting with EBNA1 on the surface opposite the DNA-binding region, where there is a large acidic patch in EBNA1 that is unique amongst the herpesvirus episome maintenance proteins. Mutagenesis of this acidic patch abolishes TRFH binding and *oriP*-dependent plasmid replication. These findings identify a previously uncharacterized acidic patch docking surface on EBNA1 essential for coordinating TRF2-RAP1 at *oriP* and provide new insights into both EBV and telomere DNA replication.

## Introduction

Epstein-Barr virus (EBV) is a double-stranded DNA herpesvirus causally linked to several B-cell and epithelial malignancies, including Hodgkin’s and Burkitt’s lymphoma, gastric carcinoma, and nasopharyngeal carcinoma^1,2^. Among the viral proteins expressed in EBV-driven tumors, Epstein-Barr Nuclear Antigen 1 (EBNA1) is the only one consistently expressed across all EBV tumor types, making it a compelling target for therapeutics intervention^3,4^. EBNA1 is essential for replicating and maintaining the viral genome in latently infected host cells. In infected cells, the EBV genome persists as circular extrachromosomal episomes within the host cell nucleus and replicates once per cell cycle from the origin of replication, or *oriP*. EBNA1 binds to a set of specific sites within *oriP* and recruits host origin recognition complex (ORC) to trigger the initiation of episome replication^5,6^. However, the precise mechanism by which EBNA1 orchestrates viral DNA replication remains incompletely understood.

Structurally, EBNA1 comprises a well-defined, sequence-specific DNA-binding domain (DBD) at its C-terminus and a less structured N-terminal that mediates diverse functions, including tethering EBV episomes to host chromosomes for nonrandom partitioning into daughter cells during mitosis^7–9^. The DBD binds to multiple sites on both viral and host DNA^10,11^ and, at *oriP*, specifically engages two elements: the *family of repeats* (FR) and the dyad symmetry (DS) regions. EBNA1 binding at FR is required for episome maintenance and segregation of daughter cells during mitosis^12^, whereas binding at DS establishes a functional origin of replication by recruiting ORC and the MCM helicase complex^5,6,13–15^.

The DS region also harbors three nonamer motifs (TTAGGGTTA), that recruit the shelterin components TRF1 and TRF2^16,17^. These proteins, canonically telomere protectors, alternately associate with *oriP* and regulate EBV replication in an EBNA1- and cell-cycle-dependent manner^16,18^. Perturbation of these sites—through mutation of the telomeric nonamers^16,17^ or knockdown of TRF2 or Rap1—impairs oriP replication, underscoring the importance of telomeric factors into EBV’s replication strategy^17,19^. Multiple studies have shown that TRF2 is involved in the regulation of DNA replication at the telomere as well as at the EBV *oriP*, with both the N-terminal basic tail and the dimerization domain of TRF2 directly implicated in ORC recruitment^20–24^.

The EBNA1 DBD has been structurally characterized alone and in complex with the minimal replicative unit of the DS (“½ DS”), by both cryo-electron microscopy (cryo-EM) and X-ray crystallography^25–27^. Building on these foundations, we employed cryo-EM, cross-linking mass spectrometry (XLMS), and Alphafold3 modeling to investigate the complex of EBNA1 DBD with ½ DS in the presence of TRF2 and its binding partner Rap1. Our results reveal a dynamic, flexible complex in which a unique acidic patch on EBNA1 stabilizes TRF2 and Rap1 at *oriP*, providing insight into how EBNA1 cooperates with telomere-binding factors to initiate EBV replication.

## Results

### TRF2, Rap1, and the EBNA1 DBD form a stable but highly flexible complex with ½ DS.

**Figure 1:**
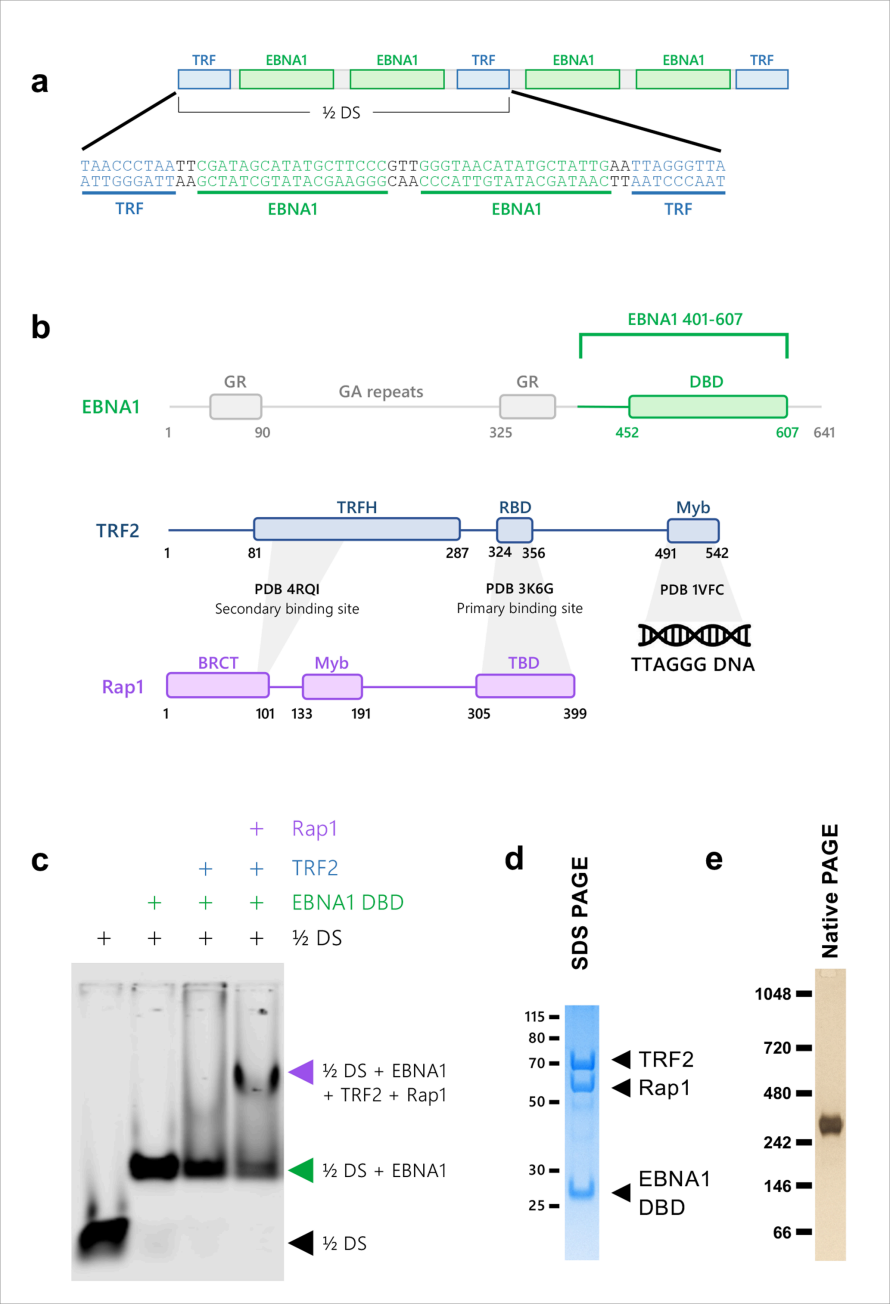
Formation of a stable TRF2-Rap1-EBNA1-½DS complex with purified components. **a**, Binding sites for EBNA1 are flanked by TTAGGGTTA sequences in the ½DS, a minimal origin of replication in EBV. **b**, Domain architecture of EBNA1, TRF2, and Rap1. Known characterized interactions are highlighted. Abbreviations: TRFH, TRF2 homodimerization domain. RBD, Rap1-binding domain. BRCT, BRCA1-like C terminal domain. TBD, TRF2-binding domain. **c**, Electrophoretic mobility shift assay (EMSA) shows cooperative binding of TRF2 and Rap1 to the ½DS in the presence of EBNA1. **d**, SDS PAGE of complex protein components EBNA1 DBD (401–607), full length TRF2, and full length Rap1. **e**, Silver stained native PAGE of final ½DS, EBNA1 DBD, TRF2, and Rap1 complex purified by glycerol gradient with glutaraldehyde fixation.

To obtain structural insight into the EBNA1-TRF2-Rap1-*oriP* complex, we first assessed the assembly of the complex by electrophoretic mobility shift assay (EMSA) using the 60 bp minimal origin of replication from DS (½ DS) containing 2 EBNA1 binding sites flanked by a single nonamer telomere repeat (Fig. 1a). The EBNA1 DNA binding domain (DBD) aa 401–607, full-length TRF2, and full-length Rap1 were expressed in *E. coli* separately and purified to near homogeneity. As expected, binding of the EBNA1 DBD to the ½ DS yielded a single discrete bandshift, indicating stable complex formation. Addition of TRF2 to the pre-formed ½ DS-EBNA1 complex yielded an unstable supershift, that appeared as a diffuse smear, whereas inclusion of Rap1 converted that smear into a stronger, more discrete supershift. This is consistent with Rap1 stabilizing TRF2-EBNA1-½ DS complex (Fig. 1c).

Because the EMSA results suggested a stable core, but potential heterogeneity in the larger assembly, we used cryo-EM to characterize the architecture of EBNA1 DBD, full-length TRF2, and full-length Rap1 assembled on ½ DS. The complex was purified using the GraFix method^28^ and monodisperse fractions as assessed by Native PAGE were selected for single particle cryo-EM analysis (Fig. 1d–e and Supplementary Fig. 1a-e). Initial 2D classification revealed a well-defined density coincident with the previously solved ½ DS EBNA1 DBD structure, while much of the surrounding protein was heterogeneous, indicating extensive conformational flexibility.

**Figure 2:**
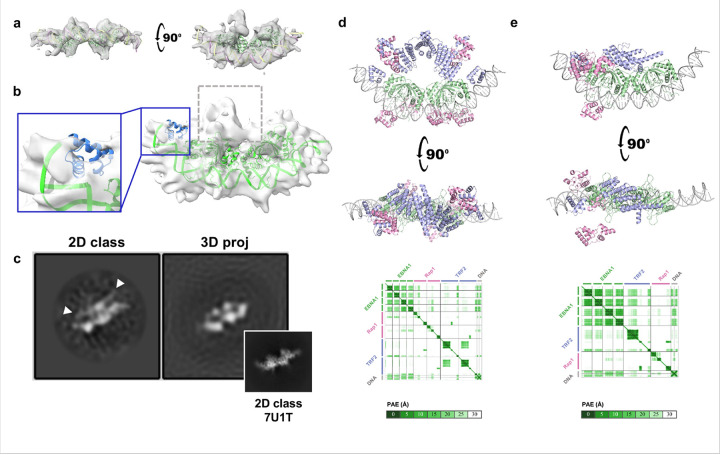
Cryo-EM and Alphafold3 together suggest that the TRF2 dimerization domain interacts with EBNA1 on the opposite side of the DNA-binding groove. **a**, 7.1 Å cryo-EM map of the ½DS + EBNA1 DBD + TRF2 + Rap1 complex at low signal (gray) shows distinct density matching up with the ½DS DNA and EBNA1 DBD (green). **b**, At higher signal threshold, the 3D model shows limited densities that likely represent flexible regions of TRF2 or Rap1. One density matches up with one TRF2 Myb domain (inset) while the other does not fit well with known TRF2 or Rap1 structured domains (gray dotted box). **c**, Despite 3D models lacking a full density for the TRF2 homodimerization domain, cryo-EM 2D classification shows a region consistent with the size and shape of a full dimer above the EBNA1 ½DS complex. Comparison of 2D classification with projections from our 3D model are shown side-by-side, along with a 2D classification from the ½DS-EBNA1 complex alone, 7U1T (inset).**d**, When given the stoichiometric ratio of 1 DNA : 4 EBNA1 : 2 TRF2 : 2 Rap1, Alphafold3 consistently places the TRF2 homodimerization domain on the side of EBNA1 opposite the DNA-binding groove, but always strictly predicts perfect C2 symmetry for the whole complex. **e**, When given only one copy each of TRF2 and Rap1, Alphafold3 predicts that one monomer of the TRF2 homodimerization domain would interact with EBNA1 in a manner consistent with the cryo-EM map. This prediction aligns well with our cryo-EM map, though Rap1 remains unresolved.

After several rounds of 2D classification and *ab initio* 3D sorting in cryoSPARC, we isolated a subset of particles that produced a 7.1 Å reconstruction (Fig. 2a–b). The core of this map aligns closely with the published ½ DS-EBNA1 DBD crystal structure (PDB ID 6PW2)^26^ and the ½ DS cryo-EM structure (PDB ID 7U1T)^27^, and clearly resolves the DNA grooves (Fig. 2a). Additional peripheral density adjacent to EBNA1 likely represents the TRF2 Myb domain binding to the TTAGGGTTA nonamer binding site (Fig. 2b, inset). The largest extra density, however, sits on the dorsal surface of EBNA1 opposite the DNA-binding interface (Fig. 2b, gray dotted box). This density did not map cleanly to published structures of TRF2 or RAP1 domains, although comparisons of 2D class averages with projections showed features consistent with the TRF2 homodimerization domain (TRFH) that are largely averaged out during 3D refinement (Fig. 2c).

To test whether the TRFH-like density could be reconciled with structural models, we used Alphafold3. When given the expected stoichiometric ratio of 1 DNA : 4 EBNA1 : 2 TRF2 : 2 Rap1, Alphafold3 models consistently placed TRFH in the dorsal region, but these models all assumed perfect C2 symmetry, which our cryo-EM data did not support (Fig. 2d). Running AlphaFold3 with only a single TRF2- Rap1 unit (to break C2 symmetry) consistently positioned the TRFH domain on the EBNA1 dorsal region and produced orientations that matched several features of the 7.1 Å cryo-EM map, including the TRFH dimerization surface (Fig. 2e, Supplementary Fig. 2). Together, cryo-EM and Alphafold3 modeling indicate a stable EBNA1-½ DS core with TRF2-Rap1 flexibly associated, and place the TRFH domain on the EBNA1 surface opposite the DNA binding region.

### XLMS reveals a concentrated EBNA1 acidic patch that contacts TRF2-Rap1.

**Figure 3:**
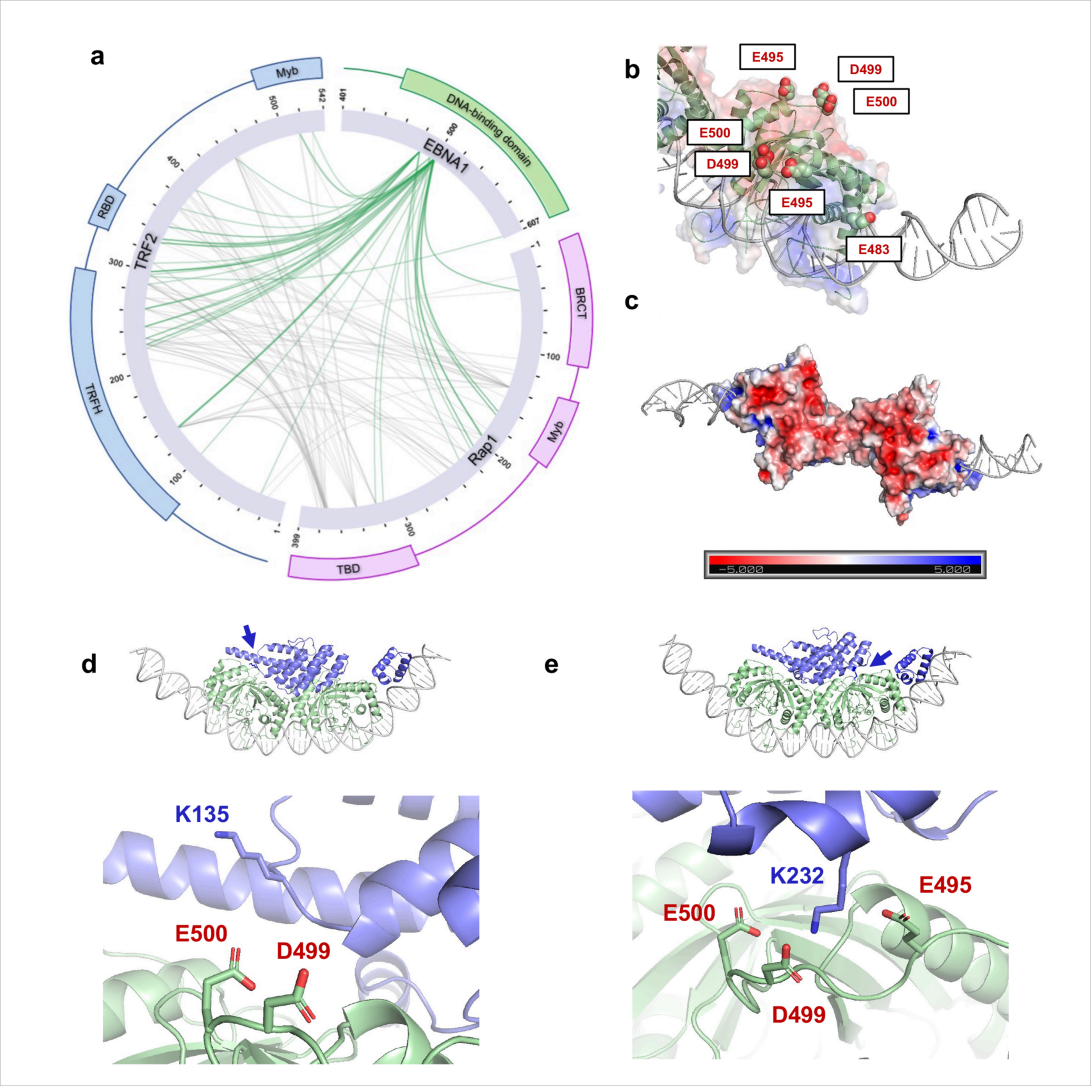
Crosslinking mass spectrometry highlights multiple zero-length interactions TRF2, Rap1, and the acidic patch of EBNA1. **a**, Intramolecular interactions between EBNA1, TRF2, and Rap1 as measured by XLMS via EDC crosslinking in complex with ½DS DNA. **b**, Acidic residues E483, E495, D499, and E500 were top hits in XLMS experiments with EBNA1, TRF2, and Rap1. **c**, E495, D499, and E500 are 3 of 8 residues that make up an extensive acidic patch on EBNA1 on the opposite side of the DNA-binding groove. **d-e**, The Alphafold3 model shown in Figure 2e orients several top XLMS hits in close proximity, such as TRF2 K135 and K232 aligning with EBNA1 E495, D499, and E500.

Because the cryo-EM density for TRF2-Rap1 was heterogeneous, we applied zero-length cross-linking mass spectrometry (EDC, carboxyl → amine chemistry) to map proximities within the full complex. Purified complex was cross-linked and analyzed by LC-MS/MS. We detected 402 interprotein cross-links: 246 mapped between TRF2 and Rap1, 107 between EBNA1 and TRF2, and 49 between EBNA1 and Rap1 (Fig. 3a). Crosslinks involving EBNA1 were highly concentrated on four acidic EBNA1 residues—E483, E495, D499, and E500—three of which (E483, E495, D499) form a contiguous acidic patch opposite to the DNA-binding region (Fig. 3b–c).

Mapping the top XLMS restraints on the cryo-EM density and AlphaFold3 models positions the TRFH domain on top of the acidic patch, with multiple high-confidence cross-links bridging the TRFH and the EBNA1 acidic residues (Fig. 3d–e). These orthogonal data sets therefore converge on a model in which the EBNA1 acidic patch directly engages the TRF2 TRFH domain and contributes to stabilizing TRF2-Rap1 at *oriP*.

### The acidic patch on EBNA1 is required for TRF2 and Rap1 recruitment and for replication at oriP.

**Figure 4:**
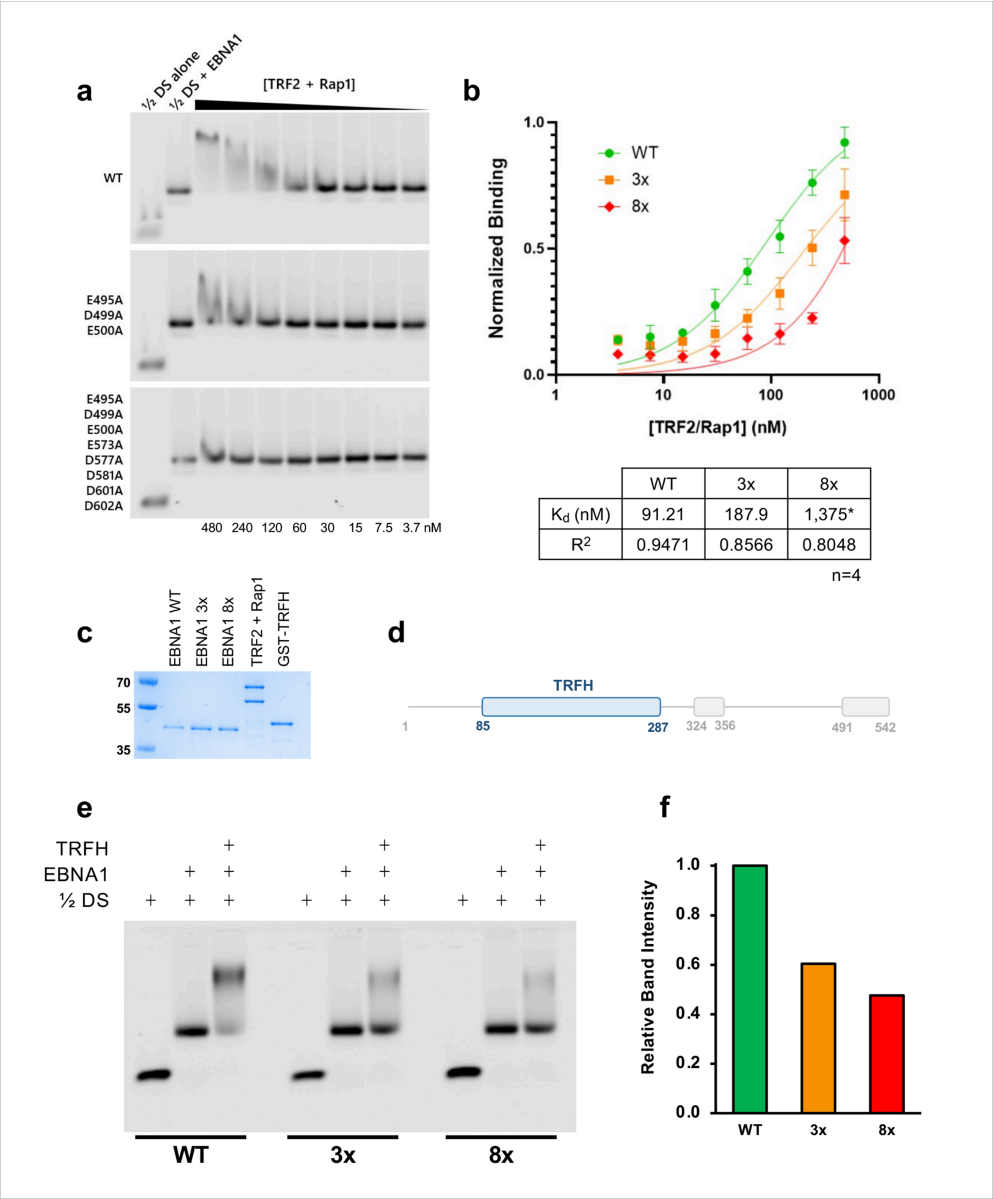
Binding of TRF2/Rap1 to the dyad symmetry element is affected by the EBNA1 acidic patch. **a**, Representative EMSA showing the binding of a full length TRF2/Rap1 complex to a pre-assembled ½DS-EBNA1 complex; binding is shown for WT EBNA1 and two acidic patch mutants. Concentration of the TRF2/Rap1 complex in nM is listed at the bottom. **b**, Quantification of TRF2/Rap1 binding to ½DS with WT EBNA1, “3x” mutant (E495A, D499A, E500A), and “8x” mutant (E495A, D499A, E500A, E573A, D577A, D581A, D601A, D602A) as measured by EMSA. Prism was able to confidently calculate Kd for WT and 3x, but not 8x, as indicated by (*). **c**, Proteins used in EMSA experiments as shown by SDS PAGE. **d**, The TRFH domain comprises residues 85–287 of TRF2. **e**, Binding of excess TRFH domain alone to the ½ DS-EBNA1 complex; binding becomes progressively weaker as more of the EBNA1 acidic patch is mutated out. **f**, Quantification of binding in **(e)**.

To determine whether the EBNA1 acidic patch is functionally required for binding of TRF2 and Rap1 to the ½ DS complex, we generated acidic-patch mutants in a Δ90–325 background (retaining all functional domains except the GA repeats: 3x (E495A D499A E500A) and an expanded mutant, 8x (E495A D499A E500A E573A D577A D581A D601A D602A). EMSA experiments showed that binding of a pre-assembled TRF2-Rap1 complex to a pre-assembled ½ DS EBNA1 complex was ~ 2 fold weaker with the 3x mutant EBNA1 and nearly undetectable for the 8x mutant EBNA1 (Fig. 4a–b), indicating the acidic patch substantially contributes to complex stability.

We next asked whether the TRFH domain alone is sufficient to engage the acidic patch. The isolated TRFH domain (Fig. 4c–d) produced a weak supershift under standard conditions, but under low salt and higher protein concentration conditions a clear supershift was observed. Importantly, the TRFH supershift was progressively reduced for the 3x and largely abolished for the 8x mutant of EBNA1 (Fig. 4e–f). The TRFH interaction did not require domains outside the DBD: a clear supershift was observed using EBNA1 401–607 and with a shorter EBNA1 459–607 that contains only the core structured DBD (Supplementary Fig. 3). These data indicate that the acidic patch on the EBNA1 dorsal surface is a direct—and functionally important—contact site for the TRFH domain.

**Figure 5:**
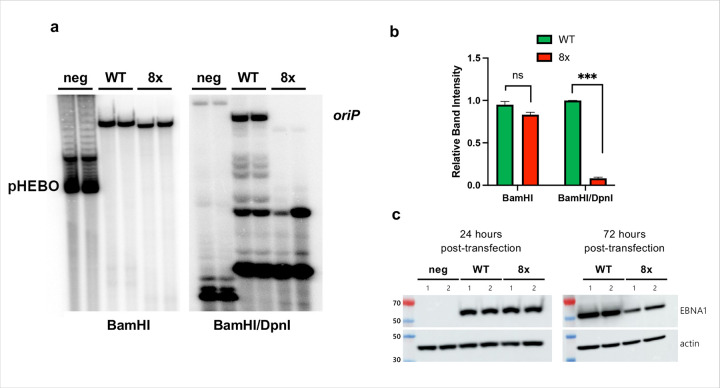
The EBNA1 acidic patch is essential for OriP-dependent replication. **a**, oriP plasmid DNA replication assay shows difference in plasmid replication over a 72 hour period between a negative control sample (no EBNA1), WT EBNA1, and the 8x mutant EBNA1. **b**, Quantification of BamHI and BamHI/DpnI resistant band intensity in **(a). c**, Western blot shows quantity of WT and 8x mutant EBNA1 in HEK293 cells used for the replication assay in **(a)**.

Finally, to determine whether disruption of this interface affects *oriP* replication, we compared EBNA1-dependent replication in HEK293 cells expressing either the WT or the 8x EBNA1 mutant. The 8x mutant showed severe impairment of *oriP* replication relative to WT (Fig. 5a–b). Both constructs had similar expression at 24 hours post-transfection, with reduced replication in the 8x mutant occurring after 72 hours (Fig. 5c). Cycloheximide chase experiments showed comparable intrinsic protein stability for WT and 8x mutant EBNA1 over 24 h (Supplementary Fig. 4a-b), arguing against gross instability as the cause of the replication defect. Taken together with the structural data and biochemical data, these results indicate that the EBNA1 acidic patch coordinates TRF2 and Rap1 at *oriP* and is essential for *oriP*-dependent EBV replication.

## Discussion

We show that the EBNA1 DBD assembles with TRF2 and RAP1 on the minimal ½ DS origin and that an extended acidic surface on the dorsal face of the EBNA1 DBD directly stabilizes this complex. Cryo-EM, Alphafold3 modeling, and zero-length cross-linking mass spectrometry converge on a model in which TRF2 homodimerization domain engages a conserved cluster of acidic residues on EBNA1 opposite the DNA-binding interface. Cryo-EM analyses suggest there is considerable conformational flexibility in this ternary complex. Consistent with this structural model, targeted neutralization of those acidic residues on the EBNA1 markedly weakens TRF2-Rap1 recruitment *in vitro* and abolishes *oriP*-dependent replication in cells. Together, these data identify a discrete EBNA1 surface that is both necessary for ternary complex formation and essential for EBV *oriP* function.

**Figure 6:**
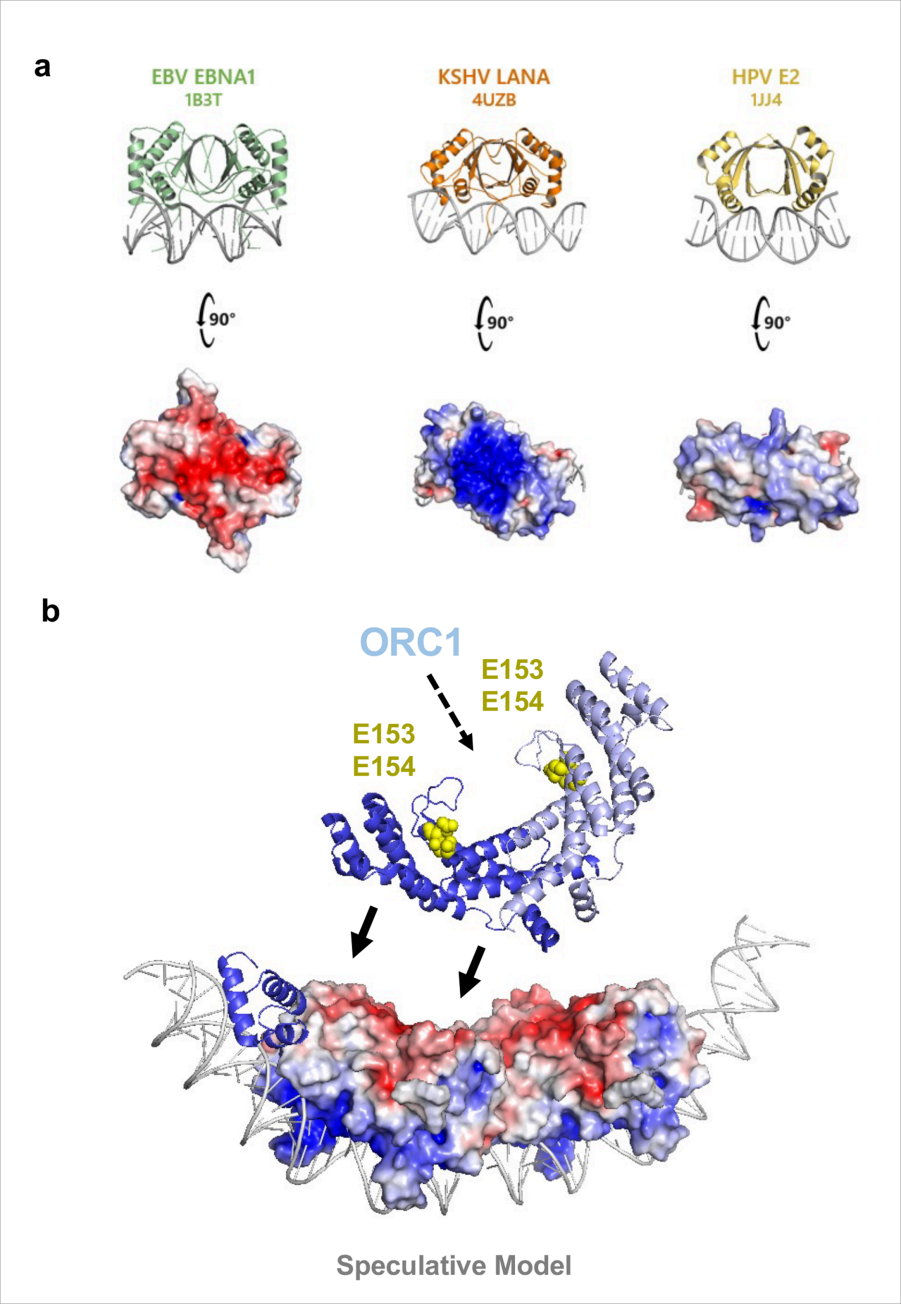
The EBNA1 acidic patch is unique amongst the herpesvirus episome maintenance proteins and ideally positioned for ORC recruitment to oriP. **a**, EBV EBNA1 (PDB:1B3T), Kaposi’s sarcoma herpesvirus LANA (PDB:4UZB), and human papillomavirus E2 (PDB:1JJ4) share structural and functional homology, but only EBNA1 possesses an acidic patch on the face opposite the DNA-binding groove. **b**, Model showing the predicted mechanism of TRF2 recruiting ORC1 to *oriP* through E153 and E154 in the TRFH domain. We propose that the interaction of the TRFH domain with the EBNA1 acidic patch as presented in this work would orient the TRFH domain in a position ideal for the recruitment of ORC1.

The acidic patch on EBNA1 appears to be a distinctive feature of EBNA1 among viral episome maintenance proteins. Structurally related proteins such as Kaposi’s sarcoma herpesvirus (KSHV) LANA (PDB ID 4UZB)^29^ and human papillomavirus E2 (PDB ID 1JJ4)^30^, lack an analogous dorsal acidic surface of their DNA-binding groove (Fig. 6a). In the case of LANA, there is a basic patch implicated in interacting with BRD2/4 family members^31^ and episome maintenance in KSHV latency^32^. Shelterin components have not been reported to associate with LANA or E2 at their respective viral origins of replication. Interestingly, EBNA1 also interacts with BRD2 where it mediates transcriptional activation functions, presumably through its N-terminal domain. E2 also interacts with BRD4^33^ to mediate mitotic chromosome tethering^34,35^ through its N-terminal tethering domain and not its DNA binding domain^36^. This suggests that EBV has evolved a unique mechanism—co-opting telomere-binding factors via an acidic EBNA1 surface—to integrate telomere biology into its episome maintenance program. Such divergence may explain differences in how these viruses tether, replicate and partition their genomes during latency.

Our data also provide a mechanistic link between TRF2 and origin licensing at *oriP*. Previous studies implicate both the N-terminal basic tail of TRF2 (RNA-dependent) and the TRFH (RNA-independent) in ORC recruitment^22^. The TRFH domain’s orientation on the EBNA1 dorsal face in our models places a convex surface towards EBNA1 while leaving the concave surface—previously implicated in ORC engagement through two glutamate resides, E153 and E154^23^—accessible (Fig. 6b). This geometry offers a simple explanation for how a single TRF2 dimer bound to *oriP* could simultaneously engage EBNA1 and present an ORC-recruitment interface, and why the ½ DS, with its two TRF2 Myb binding domains, functions as a minimal replicator in EBV.

We do not claim that TRF2 is the only protein engaging the acidic patch on EBNA1. EBNA1 is a multifunctional hub that interacts with numerous cellular factors, many mapped to the N-terminal region, but with additional partners still to be mapped precisely to the DBD. For example, EBNA1 interaction partners such as USP7^37^, CK2 subunits^38,39^, Importin alpha (KPNB1)^40,41^, have been found to bind to the regions N-terminal to the DBD. The conformational flexibility we observe suggests that the EBNA1-TRF2-Rap1 complex is dynamic and may be remodeled across the cell cycle or in response to the chromatin context, allowing other factors (including chromatin regulators, RNAs such as TERRA or additional shelterin components) to transiently occupy or modulate this interface. Importantly, our mutational data demonstrate that, regardless of additional partners, the acidic patch is functionally indispensable for *oriP* replication and for stable TRF2-Rap1 association *in vitro*.

There are limitations and clear next steps. Conformational heterogeneity limited the resolution of the full complex in cryo-EM. Higher resolution structure will likely require strategies to reduce mobility (for example, engineered cross-links, stabilizing binding partners, or in-cell crosslinking approaches). Functionally, it will be important to test whether EBNA1-TRFH contacts directly promote ORC recruitment in cells (for example, by ChIP for ORC components on *oriP* in the presence of WT versus acidic-patch mutants), to map the dynamics of TRF1/TRF2/Tankyrase exchange at DS through the cell cycle, and to probe the role of TERRA and RNA in modulating these interactions^22 42,43,44,45^. Finally, because the acidic patch is conserved across EBNA1 variants, but absent from other viral DBDs, it represents a plausible, specific target for therapeutic disruption of EBV episome maintenance. Screening for small molecules or peptides that block EBNA1-TRFH interface is therefore an attractive translational direction.

In summary, our integrated structural and functional analyses identify a conserved acidic surface on the EBNA1 DBD as a central coordinator of TRF2-Rap1 recruitment to *oriP* and provide a mechanistic framework linking shelterin components to EBV origin function. These findings expand our understanding of how viral and telomeric factors cooperate to initiate latent viral DNA replication and open concrete routes for further mechanistic and therapeutic investigation.

## Methods

### Plasmids and mutagenesis:

Constructs encoding EBNA1 (DBD and longer constructs), full-length TRF2 and full-length Rap1 were cloned into pET expression vectors with an N-terminal His6–SUMO tag using BamHI and SalI restriction sites. The EBNA1 constructs used in this study were: the DNA-binding domain (DBD) EBNA1(401–607) and an N-terminal deletion construct EBNA1ΔGA (Δ90–325) — the latter retains all functional domains except the glycine–alanine repeat region and is referred to in the text as “full-length” EBNA1 for functional assays. EBNA1Δ90–325 3x (E495A D499A E500A) and 8x (E495A D499A E500A E573A D577A D581A D601A D602A) acidic-patch mutants were generated by site-directed mutagenesis (New England Biolabs Q5 protocol) and sequence-verified.

### Protein expression

All constructs were expressed in *E. coli* using autoinduction medium for 24 h at 22°C, as described previously^26^.

#### Protein purification:

Cells were lysed using lysozyme and sonicated in a lysis buffer with 1% Tween and PMSF and clarified by centrifugation. The His-SUMO-tagged proteins were purified on Ni-NTA beads (Genesee Scientific) and eluted with buffer containing 300 mM imidazole. Proteins were concentrated and subjected to size-exclusion chromatography on a HiLoad 26/60 Superdex 75 gel filtration column (Cytiva). Tags were removed enzymatically: SUMO tags cleaved with ULP1 protease and constructs containing a TEV site were cleaved with TEV protease. After protease digestion, samples were passed over Ni-NTA resin a second time to remove uncleaved His-SUMO and His-tagged protease. Final polishing was performed by a second Superdex 75 run. Fractions corresponding to monodisperse protein were pooled, concentrated (typically > 2 mg/ml), flash-aliquoted and stored at −80° C. Protein purity and integrity were assessed by SDS-PAGE.

### DNA substrates

The ½DS oligonucleotide duplex used for biochemical and structural studies was purchase from IDT, Inc (5’-TAACCCTAATTCGATAGCA TATGCTTCCCGTTGGGTAACATA TGCTATTGAATTAGGGTTAG-3’; complementary strand synthesized accordingly). Duplexes were annealed by heating to 95° C for 5 min and slow cooling to 4°C over 2.5 h.

#### Complex assembly and glycerol-gradient purification:

Protein-DNA complexes were mixed based on the molar ratio of 1 DNA : 4 EBNA1 401–607 : 2.5 TRF2 : 2.5 Rap1 and loaded onto a glycerol gradient (10% to 30% glycerol) and centrifuged at 45,000 rpm for 16 h. Fractions were checked using both SDS and Native PAGE, and the fractions with the correct molecular weight were collected, concentrated to 1mg/mL for each sample, and buffer exchanged into 20mM MES pH 6.8, 100mM NaCl, 1mM MgCl_2_, 10μM ZnCl_2_, 0.5mM TCEP. In a 50μL reaction volume, 1mg/mL complex was combined with 1μL of freshly prepared 0.5M Pierce^™^ EDC, 0.25M Sulfo-NHS (N-hydroxysulfosuccinimide) (Thermo Scientific^™^) for a final crosslinker concentration of 10 mM and incubated at room temperature for 2 hours on an end-over-end rotator. Samples were quenched with 20mM DTT for 5 minutes.

### Cross-linking mass spectrometry (XL-MS)

Glycerol-gradient-purified complexes were buffer-exchanged into 20mM MES pH 6.8, 100mM NaCl, 1mM MgCl_2_, 10μM ZnCl_2_, 0.5mM TCEP and adjusted to 0.5 mg/mL in a 50μL reaction volume. Freshly prepared crosslinking reagents were added to yield final concentrations of 10mM EDC. Reactions were incubated at room temperature for 2 h on an end-over-end rotator and then quenched with 20 mM DTT for 5 min. Cross-linked samples were processed for XL-MS using established in-solution digestion and peptide-enrichment protocols (reduction, alkylation, tryptic digest, desalting) and analyzed by LC-MS/MS. Cross-link identification was performed using standard cross-linking search pipelines.

#### Cryo-electron microscopy grid preparation and data collection:

Protein-DNA complexes were mixed based on the molar ratio of 1 DNA : 4 EBNA1 : 2.5 TRF2 : 2.5 Rap1 and loaded onto a glycerol gradient (10% to 30% glycerol, with 0.125% glutaraldehyde for fixation in the 30% solution) and centrifuged at 45,000 rpm for 16 h. Fractions were checked using Native PAGE, and the fractions with the correct molecular weight were collected and concentrated to ~ 2 mg/mL for each sample. Samples were diluted to 0.5 mg/mL to apply to grids (C-flat Cu, CF-2/1–2C) using a Vitrobot Mark IV (FEI). Two rounds of cryo-EM data of the ½ DS EBNA1 TRF2 Rap1 complex were collected at the National Cancer Institute’s National Cryo-EM Facility at the Frederick National Laboratory for Cancer Research (NCI Frederick) Krios equipped with a K3 camera, both at a magnification of 81,000 (pixel size of 1.07 Å).

### Cryo-EM data processing

Motion correction, contrast transfer function (CTF) estimation, particle picking (blot picker), and 2D class were performed in cryoSPARC^46^. After several runs of 2D classification, selected good particles were pooled together and run through an *ab initio* job in cryoSPARC to yield multiple models (Class Similarity set to 0). The best model from this job and its respective particles were again split into multiple classes via the *ab initio* job, yielding one selected class with 242,948 particles. After more rounds of 2D classification to bring the number of particles down to 40,377, particles were used to create one *ab initio* model, which was then refined by homogenous refinement.

### EMSA

Protein-DNA binding reactions were assembled in binding buffer (10 mM HEPES pH 7.5, 300 mM KCl, 5 mM MgCl_2_, 1 mM ZnCl_2_, 5 mM β-mercaptoethanol [BME], 0.05% NP-40, 5% glycerol) in PCR tubes. For the protein being measured, a serial dilution of 5 μL 10X concentration protein was created, and to each tube, 40 μL binding buffer and 5 μL 50 nM DNA probe were added for a final concentration of 5nM DNA probe and final reaction volume of 50 μL. Probes for 60 bp DS DNA were synthesized with 5’-IRD700 dye on both strands (IDT). 15 μL of each sample were added into each well of a 1.4% agarose gel, and 90 constant voltage was applied for 1.5 h. The gel was imaged using the LI-COR imaging system. For binding reactions of TRF2/Rap1 to the ½ DS EBNA1 complex, pre-assembled TRF2-Rap1 complex was purified by glycerol gradient and then serial diluted in 5 μL volumes as above. To this, 40μL of binding buffer and 5 μL of a 10X stock solution of 200nM EBNA1 and 50nM ½ DS DNA was added. For the binding reactions of the TRF2 dimerization domain (TRFH) to the ½ DS EBNA1 complex, the binding buffer was lowered to 100 mM KCl to encourage electrostatic interactions, and the highest concentration of TRFH was 10 μM (1000-fold stoichiometric excess).

#### Cyclohexamide chase experiments:

HEK293T cells were either transfected with an empty vector (N1063), wild-type flag-tagged EBNA1 (WT-FLAG-EBNA1) (N2624), or a mutant EBNA1 with the acidic patches substituted (Δ8X-FLAG-EBNA1) (N3739), then treated with cycloheximide (ThermoFisher-Cat:J66004.XF) as indicated in Supplementary Fig. 4.

### Replication Assay

HEK293 cells were transfected with plasmids encoding N-terminal FLAG-tagged EBNA1 (WT or 8x mutant) expressed from a CMV promoter on an oriP-containing, hygromycin-resistance backbone. Seventy-two hours post-transfection, low-molecular-weight DNA was prepared by Hirt lysis, digested with BamHI or BamHI + DpnI, separated by agarose gel electrophoresis and analyzed by Southern blot using an *oriP* probe, as previously described^47^. Protein expression and stability were assessed by Western blot; cycloheximide chase experiments were performed to compare protein stability between WT and mutant EBNA1

## Supplementary Files

This is a list of supplementary files associated with this preprint. Click to download.

- SupplementaryMaterialsEBNA1TRF2Rap109092025plv6.pdf
- EBNA1TRF2Rap1CryoEMStats17604763291.doc
- StructBasisTRF2Rap1EBNA1.zip
